# SIRT2 is an unfavorable prognostic biomarker in patients with acute myeloid leukemia

**DOI:** 10.1038/srep27694

**Published:** 2016-06-13

**Authors:** Ailing Deng, Qiaoyang Ning, Lei Zhou, Yaojie Liang

**Affiliations:** 1 Medicine College, Nankai University, Tianjin, 300071, China; 2 Department of Hematology, No.202 Hospital of PLA, Shenyang, 110083, China.; 3 Department of Senior Hematology and Oncology, the First Affiliated Hospital of Chinese PLA General Hospital, Beijing, 100048, China

## Abstract

SIRT2 is a member of the NAD+ dependent deacetylases. In this study, the associations between SIRT2 expression and molecular and clinical characteristics of patients with acute myeloid leukemia (AML) were evaluated by data from The Cancer Genome Atlas. SIRT2 was overexpressed in the intermediate- and poor-risk groups of patients, compared to the favorable-risk group of patients (*P* = 0.002 and 0.004, respectively). High SIRT2 expression was associated with significantly shorter overall survival (OS; *P* = 0.0005) and event-free survival (EFS; *P* = 0.0002) than low SIRT2 expressio in a cohort of 167 patients with AML. Multivariate analyses revealed that high SIRT2 expression was associated with shorter OS (*P* = 0.031) and EFS (*P* = 0.020). Gene-expression profiling showed 259 differential expressed genes including CD4, CD14 and IL10. Gene sets like MAPK signaling pathway, VEGF signaling pathway and acute myeloid leukemia were upregulated in SIRT2^high^ patients. We also found different methylation patterns in these two groups. OS and EFS of SIRT2^high^ patients who did not undergo transplantation were significantly shorter than those of SIRT2^low^ patients (*P* = 0.0120 and *P* = 0.0107, respectively). Taken together, these findings suggest that high SIRT2 expression is a novel and unfavorable prognostic biomarker for AML risk-stratification.

Acute myeloid leukemia (AML) is a group of heterogeneous hematopoietic stem cell disorders^1^. Many recurrent chromosomal structural variations and mutations contribute to AML pathogenesis, such as the AML1-ETO, PML-RARA, FLT3 and DNMT3A mutations^2^,^3^,^4^. However, as the biological features of AML are elucidated, epigenetic lesions contributing to the generation of AML becomes evident^5^,^6^.

SIRT2 is a member of the sirtuin family which deacetylates lysines on histone and alpha-tubulin as well as many proteins such as key transcriptional factors P53, NF-κB and so on^7^. SIRT2 has five isoforms and is mainly involved in NAD metabolism and chromatin regulation or acetylation. It participates in the modulation of multiple biological processes such as cell cycle control, genomic integrity, microtubule dynamics, cell differentiation, DNA repair, metabolic networks, autophagy and pathological processes such as tumorigenesis, neurodegeneration, survival and drug resistance of cancer cells^8^,^9^,^10^,^11^,^12^. SIRT2 is the primary cytoplasmic surtuin but shuttles continuously between cytoplasmic and nuclear comparts during interphase, and it is found to be involved in the proliferation and survival of acute myeloid leukemia. Levels of SIRT2 mRNA significantly elevated in AML blasts compared to levels in bone marrow from healthy individuals and in a high-risk group pf AML patients, it is significantly higher than that in a standard-risk group^13^. FAB subtypes M1, M2 and M4 patients have lower levels of SIRT2 while M5 patients have higher levels. What’s more, SIRT2 is expressed at higher level in the relapsed AML patients than newly diagnosed patients^14^. Besides, SIRT2 participates in the aberrant proliferation and survival of leukemic cell, and inhibition of SIRT2 by compunds leads to induced cell cycle arrest, elevated apoptosis, reduced proliferation and granulocytic differentiation in AML^14^,^15^,^16^,^17^. It is also suggested that high SIRT2 expression leads to DNR/Ara-C resistance in AML cells through the ERK1/2 pathway^13^. What’s more, some studies also demonstrate that inhibiting SIRT2 with compounds acetylate and activate the tumor suppressor TP53, which is critical for controlling cell growth and apoptosis during cellular stress, and mutations of which are an unfavorable prognostic factor in patients with AML^18^,^19^. So, it is hypothesized that SIRT2 may be related to the survival of AML and participate in leukemogenesis of it, so we downloaded data from The Cancer Genome Atlas (TCGA) to evaluate the associations between SIRT2 expression and molecular and clinical characteristics.

## Methods

### Patients and treatment

Patients with complete data (clinical, RNASeqV2, miRNASeq, somatic mutations and humanmethylation450) were included in this article. A total of 167 patients with previously untreated AML (median age, 58 years; range: 18–88 years) were studied, all of whom had been diagnosed and received treatment according to the National Comprehensive Cancer Network (NCCN) guidelines between November 2001 and March 2010^18^. In total, 87 patients (52.1%) were aged <60 years (younger patients) and 80 patients (47.9%) were ≥60 years (older patients). The diagnosis and risk stratification of AML were based on the NCCN Guidelines. All patients were assessed for somatic mutations, such as IDH1, NPM1, FLT3, and gene expression. Clinical, gene and miRNA expression, methylation and somatic mutation profiles of all primary AML cases were publicly downloaded from the TCGA project via the data portal on January 10, 2015.

### Gene expression analyses

Methylation, RNA and miRNA sequencing data were published previously and downloaded from the TCGA. Normalized counts were calculated to represent the normalized expression levels of either the genes or miRNAs. We subdivided the 167 AML patients into four quartiles based on SIRT2 expression level, and patients with SIRT2 Q4 (75–100%) expression values were subdivided into a SIRT2^high^ group while the others were in the SIRT2^low^ group.

### Statistical analyses

The time from date of diagnosis to removal from the study due to the absence of complete remission, relapse, or death defined event-free survival (EFS), and the time from the date of diagnosis to death due to any cause defined overall survival (OS). The Gehan–Breslow–Wilcoxon test was used to estimate the association between SIRT2 expression and EFS and OS of the patients, which was further validated using the log-rank test. To investigate the associations between SIRT2 expression levels and the clinical and molecular characteristics, the Fisher’s exact and Wilcoxon rank-sum tests were used in the hypothesis testing for categorical and continuous variables, respectively. Student’s *t*-test and multiple hypothesis correction (false discovery rate) were used to identify differences in gene expression profiles between the SIRT2 expression level groups. The statistical cutoff values were a fold-change ≥1.5 and an adjusted p-value ≤0.05. All analyses were performed using the R (ver. 3.2.2; R Core Development Team, Vienna, Austria) and GraphPad Prism software packages (ver. 5.0; GraphPad Software Inc., La Jolla, CA, USA).

## Results

### SIRT2 expression in AML samples

**Figure 1:**
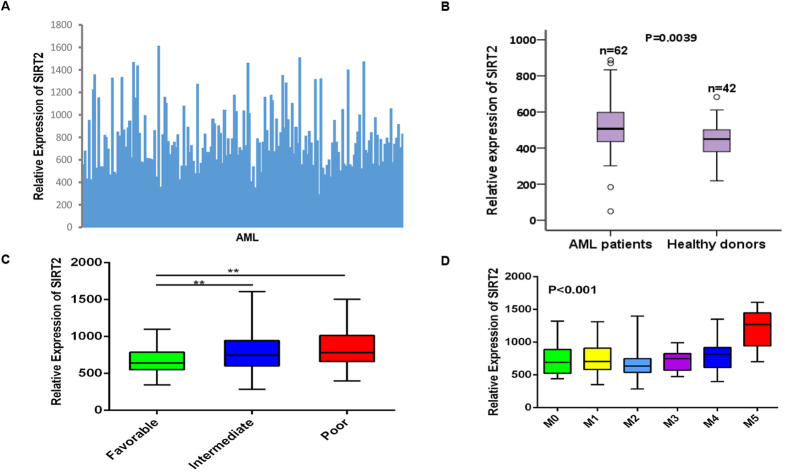
SIRT2 expression in patients with acute myeloid leukemia (AML). (**A**) Relative expression of SIRT2 in 167 patients with AML. (**B**) Relative expression of SIRT2 in 62 AML cases compared with 42 normal bone marrow samples. (**C**) Relative expression of SIRT2 in the different NCCN subtypes. (**D**) Relative expression of SIRT2 in the different FAB subtypes. **P < 0.01.

RNASeqV2 data were downloaded from the TCGA database and analyzed to obtain the SIRT2 expression levels for all AML patients. As indicated in Fig. 1A, SIRT2 was highly expressed in most patients with AML.

### Association between SIRT2 expression level and the primary patient characteristics

We analyzed SIRT2 expression using a microarray assay, and there was also a significantly higher bone marrow SIRT2 expression in AML patients than normal donors (*P* = 0.0039; Fig. 1B) (GEO accession number GSE63270). Of the 167 patients, SIRT2 expression was higher in the NCCN poor- and intermediate-risk patients than that in the favorable-risk group (*P* = 0.002 and 0.004, respectively; Fig. 1C). Those with subtype M5 showed the highest SIRT2 expression level, whereas those with the M2 subtype had lower SIRT2 expression levels (*P* < 0.001, Fig. 1D). The M5 group in the FAB subtype was more likely to have SIRT2^high^ patients, whereas the M2 group was not (*P* < 0.001 and *P* = 0.028, respectively; Table 1). In addition, the number of SIRT2^high^ and SIRT2^low^ patients was significantly different in the NCCN-favorable group (P = 0.012). No association was detected between SIRT2 expression and any other single mutation, such as FLT3 or NPM1. No differences in sex or pre-treatment white blood cell (WBC) count, hemoglobin, platelet count, or blast percentage in the bone marrow were detected between these two groups, whereas patient age in the SIRT2^high^ group were higher than those in the SIRT2^low^ group (*P* = 0.032; Table 1).

### High SIRT2 expression is an unfavorable factor in patients with AML

**Figure 2:**
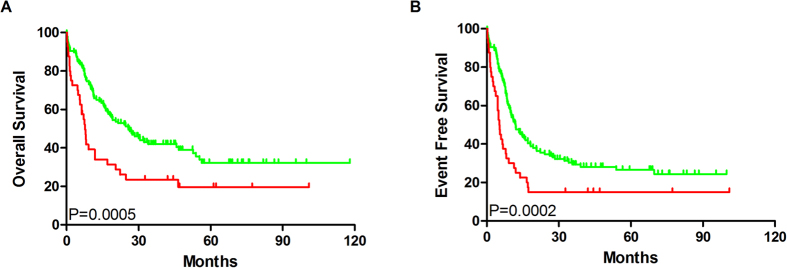
High SIRT2 expression is associated with an unfavorable prognosis. (**A**) Overall survival (OS) and (**B**) event-free survival (EFS) in the cohort of 167 AML cases. Red line: SIRT2^high^ group (n = 41); green line: SIRT2^low^ group (n = 126).

**Figure 3:**
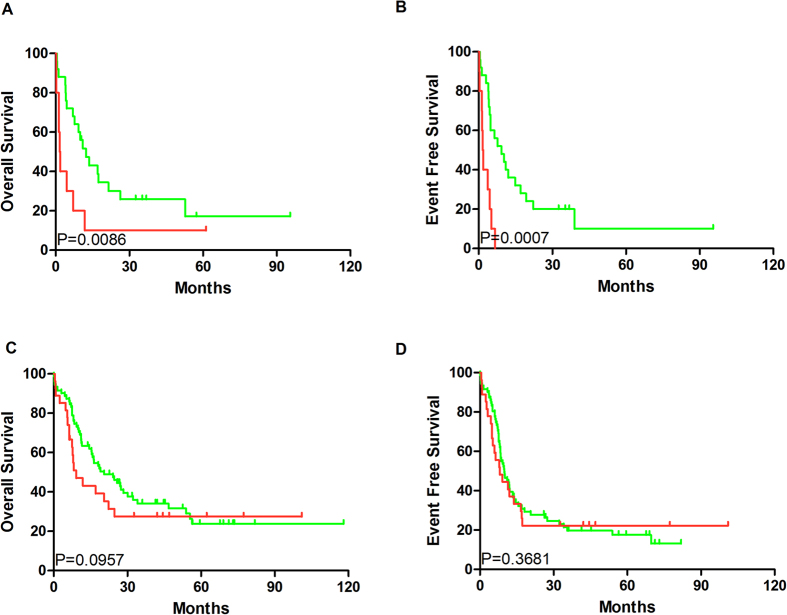
Variable prognosis associated with different SIRT2 expression in the poor-risk and intermediate-risk groups. (**A**) OS and (**B**) EFS in the poor-risk group (SIRT2^high^ group, n = 11; SIRT2^low^ group, n = 25); (**C**) OS and (**D**) EFS in the intermediate-risk group (SIRT2^high^ group, n = 27; SIRT2^low^ group, n = 71). Red line: SIRT2^high^ group; green line: SIRT2^low^ group.

Median OS and EFS of the SIRT2^high^ group (7.7 and 25.8 months and 5.25 and 12 months, respectively) were significantly shorter than those of SIRT2^low^ patients (*P* = 0.0005; *P* = 0.0002, respectively; Fig. 2), according to Gehan–Breslow–Wilcoxon test, which were then revalidated by the log-rank test. Furthermore, high SIRT2 expression in poor-risk patients was associated with shorter EFS and OS (Fig. 3A,B). No differences in EFS and OS were detected in the intermediate-risk subgroup (Fig. 3C,D).

After adjusting for the effect of several known risk factors, we performed multivariate analyses to determine the prognostic significance of SIRT2 expression. The SIRT2^high^ group had shorter OS and EFS in the multivariate model (*P* = 0.031, *P* = 0.020, respectively; Table 2). Poor cytogenetic classification, age were also associated with shorter OS and EFS.

### Genome-wide gene-expression profiles associated with SIRT2 expression

**Figure 4:**
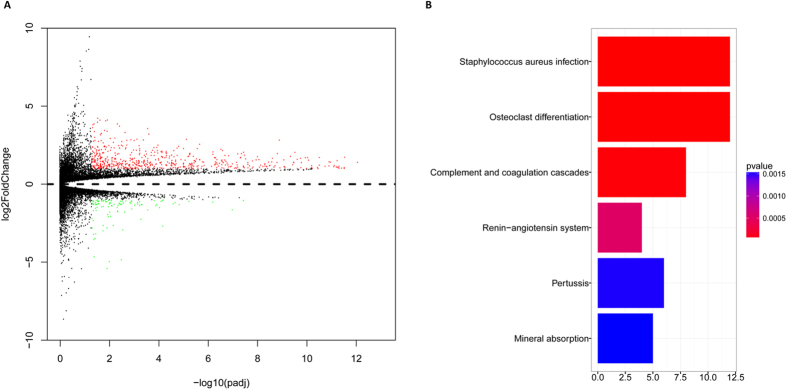
Gene expression analysis associated with SIRT2 expression. (**A**) Expression volcano map of associated genes. (**B**) KEGG pathway analysis. Number of genes involved in the indicated pathway was plotted as abscissa.

We derived SIRT2-associated gene expression profiles to further evaluate the role of SIRT2 in patients with AML. We identified 228 upregulated genes and 31 downregulated genes that were significantly associated with high SIRT2 expression (Fig. 4A, Supplemental Tables S1 and S2). The upregulated genes included some of those previously found to be involved in AML, including members of the heat shock proteins (HSPA6 and HSPA7), and those involved with normal differentiation of monocytes/macrophages, such as CEBPB, and immune functions, such as CD4, CD14 and IL10. The downregulated genes included KIT (Supplemental Fig. S1). The SIRT2-associated cell signaling pathways were evaluated by KEGG pathway analysis to assess the biological features of the SIRT2 expression profile. Signaling pathways such as renin-angiotensin, complement and coagulation cascades was involved in SIRT2 pathogenicity (*P* < 0.001; Fig. 4B). The correlation network of 259 differentially expressed genes and SIRT2 indicated that SIRT2 was on the edge of molecular network center (Supplemental Fig. S2A). 31 0f 259 differentially expressed genes were closely related to the expression of SIRT2, including IL4I1, RRAS, CEBPB, KIT (Supplemental Fig. S2B and Table S3). 30 of 31 genes were positively correlated to SIRT2, while KIT was negatively correlated (Supplemental Fig. S3). By GSEA analysis, 46 gene sets were found to be significantly upregulated in SIRT2^high^ patients, including MAPK signaling pathway, VEGF signaling pathway, acute myeloid leukemia (Supplemental Table S4). A differential miRNA analysis did not reveal any differentially expressed miRNAs between these two groups. However, we found that they had different methylation patterns in SIRT2^high^ and SIRT2^low^ group (supplementary Table S5). KEGG pathway analysis showed that these differentially methylated genes participated in the TNF pathway and so on (supplementary Table S6).

### Different treatment outcomes associated with SIRT2 expression

**Figure 5:**
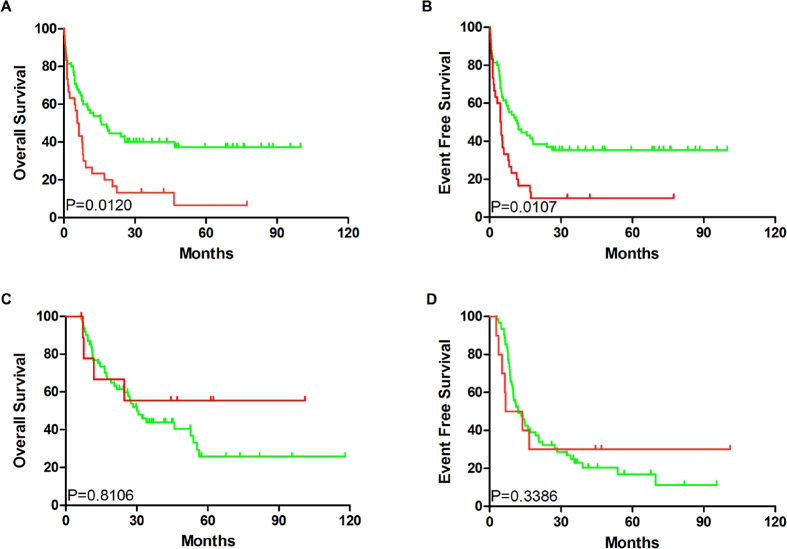
Survival analysis of the SIRT2 expression groups who did and did not undergo transplantation. (**A**) OS and (**B**) event-free survival (EFS) in patients who did not undergo transplantation (SIRT2^high^ group, n = 31; SIRT2^low^ group, n = 65); (**C**) OS and (**D**) EFS in patients who underwent transplantation (SIRT2^high^ group, n = 10; SIRT2^low^ group, n = 61). Red line: SIRT2^high^ group; green line: SIRT2^low^ group.

**Figure 6:**
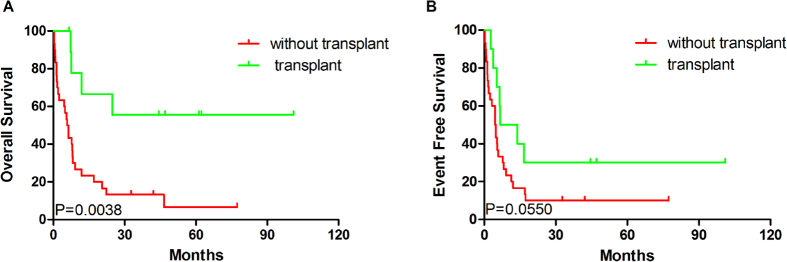
Survival analysis of patients in the SIRT2^high^ groups who did and did not undergo transplantation. (**A**) OS and (**B**) EFS of patients who did and did not undergo transplantation (without transplant group, n = 32; transplant group, n = 9).

We then analyzed the clinical outcomes of different treatment strategies associated with SIRT2 expression. As indicated in Fig. 5, OS and EFS of SIRT2^high^ patients who did not undergo transplantation were significantly shorter than those of SIRT2^low^ patients (*P* = 0.0120 and *P* = 0.0107, respectively). However, in the transplant group, OS and EFS among SIRT2^high^ and SIRT2^low^ patients who underwent transplantation were not significantly different. Transplantation prolonged OS (*P* = 0.0038), but not EFS, in SIRT2^high^ patients (Fig. 6).

## Discussion

AML is a heterogeneous disease with genetic and epigenetic lesions contributing to AML cooperatively^5^,^6^, we hypothesized that SIRT2 may be involved in leukemogenesis of AML.

Here, for the first time, we report the relationship between SIRT2 expression and prognosis of patients with AML, and found that high SIRT2 expression was associated with a poor prognosis in these patients. SIRT2 was overexpressed in the intermediate- and poor-risk groups of patients, compared to the favorable-risk group. What’s more, patients with AML and high SIRT2 expression levels had significantly shorter OS and EFS than those with low SIRT2 expression. Considering that gene expression signals are not stable due to tumor heterogeneity^20^, we used a microarray assay from NCBI and found there was a significantly higher expression of bone marrow SIRT2 in AML patients.

How did SIRT2 become associated with prognosis of AML patients? Previous studies revealed that SIRT2 regulates acetylation status and oncogenic activity of mutant KRAS. Inhibiting SIRT2 has a dramatic effect on the growth properties of cancer cells expressing KRAS activation mutants^21^. However, we found no association between SIRT2 and KRAS mutations in this article. We found that high SIRT2 expression had a tendency of high incidence of TP53 mutation while low incidence of MYH11-CBFB fusion. Taken together, different incidence rates of the MYH11-CBFB fusion gene and TP53 mutations in the SIRT2^high^ group might contribute to the poor prognosis of patients with high SIRT2 expression levels.

A differential expression analysis showed 259 differentially expressed genes associated with SIRT2 expression, signaling pathways such as renin-angiotensin, complement and coagulation cascades was involved in SIRT2 pathogenicity and Gene sets like MAPK signaling pathway, VEGF signaling pathway and acute myeloid leukemia were upregulated in SIRT2^high^ patients. Local bone marrow renin-angiotensin system functioned in the development of hematopoietic malignant disorders^22^. MAPK pathway was constitutively activated in AML blasts, suggesting a pivotal role in the process of leukemogenesis^23^,^24^,^25^. While aberrant VEGF signaling operated in leukemogenesis and was related to a poor prognosis of AML patients^26^,^27^. These might contribute to the unfavorable outcome of high SIRT2 expression. We also found that SIRT2^high^ and SIRT2^low^ AML patients had different DNA methylation patterns. In addition, SIRT2^high^ patients were more likely to have low KIT expression levels; this result is contradicted by previous studies reporting that high KIT expression levels are correlated with poor prognosis in patients with AML^28^, indicating existence of underlying mechanism in the association between SIRT2 and KIT expression.

Furthermore, we found no differences in OS or EFS between the SIRT2^high^ and SIRT2^low^ groups of patients who underwent transplantation, indicating that a transplant may improve the clinical outcomes of SIRT2^high^ patients. This was further demonstrated by the prolonged OS in the SIRT2^high^ group of patients who underwent transplantation. However, a transplant did not significantly change the EFS of the SIRT2^high^ patients.

In summary, our results confirm that a high SIRT2 expression level is related to the unfavorable prognosis of patients with AML; therefore, SIRT2 may be a new risk stratification marker, particularly for poor-risk patients with AML, and a new target for clinical therapy.

## Additional Information

**How to cite this article**: Deng, A. SIRT2 is an unfavorable prognostic biomarker in patients with acute myeloid leukemia. *Sci. Rep*. **6**, 27694; doi: 10.1038/srep27694 (2016).

## Supplementary Material

Supplementary Information

## Figures and Tables

**Table 1:** Clinical characteristics of the acute myeloid leukemia (AML) cohort according to SIRT2 expression.

Variable	SIRT2^low^(n = 126)	SIRT2^high^(n = 41)	P-value
Median age, y (range)	57 (21–82)	65 (18–88)	**0.032**
Female sex, no (%)	59 (46.8%)	22 (53.7%)	0.758
BM blast at diagnosis	68.34 ± 1.65	70.93 ± 3.23	0.452
WBC (10^9^/L)	35.69 ± 4.38	38.38 ± 5.66	0.747
Hemoglobin (g/L)	9.61 ± 0.13	9.44 ± 0.23	0.516
Platelet (10^9^/L)	63.32 ± 4.77	64.88 ± 8.18	0.306
FAB subtype, no.			
M0	12	3	0.668
M1	33	10	0.819
M2	33	4	**0.028**
M3	14	1	0.092
M4	25	7	0.696
M5	3	15	**<0.001**
M6	2	0	0.417
M7	3	0	0.319
Not Classified	1	1	0.400
NCCN subtype, no			
Favorable	28	2	**0.012**
Intermediate	71	27	0.404
Poor	25	11	0.283
Not Classified	2	1	0.721
Transplant, no (%)	61(48.4)	10(24.4)	**0.007**
Mutation, no.			
CEBPA	10	3	0.898
DNMT3A	30	11	0.696
FLT3	36	10	0.603
IDH1	13	3	0.571
IDH2	12	5	0.523
KIT	6	1	0.623
KRAS	4	2	0.611
NRAS	9	3	0.970
TP53	7	6	0.059
NPM1	33	13	0.325
FLT3+NPM1	17	7	0.570
Fusion, no			
RUNX1-RUNX1T1	6	1	0.519
PML-RARA	13	1	0.114
MYH11-CBFB	9	0	0.079

PB, peripheral blood; BM, bone marrow; NCCN, National Comprehensive Cancer Network; SIRT, sirtuin.

**Table 2:** Multivariate analysis for overall survival (OS) and event-free survival (EFS) in patients with AML.

Variable	OS, n = 167		EFS, n = 167	
	HR (95% CI)	P-value	HR (95% CI)	P-value
High SIRT2 expression	1.608 (1.045–2.473)	**0.031**	1.639 (1.080–2.486)	**0.020**
Age , per 10-y increase	1.486 (1.278–1.727)	**0.000**	1.374 (1.206–1.565)	**0.000**
Cytogenetic classification = poor	1.866 (1.243–2.802)	**0.003**	1.816 (1.265–2.608)	**0.001**
WBC > 16	1.198 (0.783–1.835)	0.405	1.562 (1.054–2.316)	0.026
RUNX1-RUNX1T1	0.830 (0.254–2.710)	0.757	0.619 (0.191–2.011)	0.425
PML-RARA	0.593 (0.229–1.534)	0.281	0.525 (0.203–1.354)	0.182
MYH11-CBFB	0.280 (0.067–1.169)	0.081	0.392 (0.139–1.105)	0.077
DNMT3A mutation	1.145 (0.732–1.789)	0.553	0.994 (0.649–1.523)	0.979
FLT3 mutation	1.504 (0.937–2.413)	0.091	1.620 (1.057–2.482)	**0.027**

HR, hazard ratio; CI, confidence interval; SIRT, sirtuin; WBC, white blood cell.
