# Supplementary material for: SIRT2 is an unfavorable prognostic biomarker in patients with acute myeloid leukemia
Source: Sci Rep. 2016 Jun 13;6:27694. doi: 10.1038/srep27694 (PMC4904374; doi:10.1038/srep27694)
Supplement: Supplementary Information [file srep27694-s1.doc]

**SIRT2 is an unfavorable prognostic biomarker in patients with acute myeloid leukemia**

Ailing Deng^1^,^*^, Qiaoyang Ning^1,*^, Lei Zhou^2^, Yaojie Liang^3^

^1^Medicine College, Nankai University, Tianjin, 300071, China.

^2^Department of Hematology, No.202 Hospital of PLA, Shenyang,110083, China.

^3^Department of Senior Hematology and Oncology, the First Affiliated Hospital of Chinese PLA General Hospital, Beijing, 100048, China.

*These authors contributed equally to this work.

**
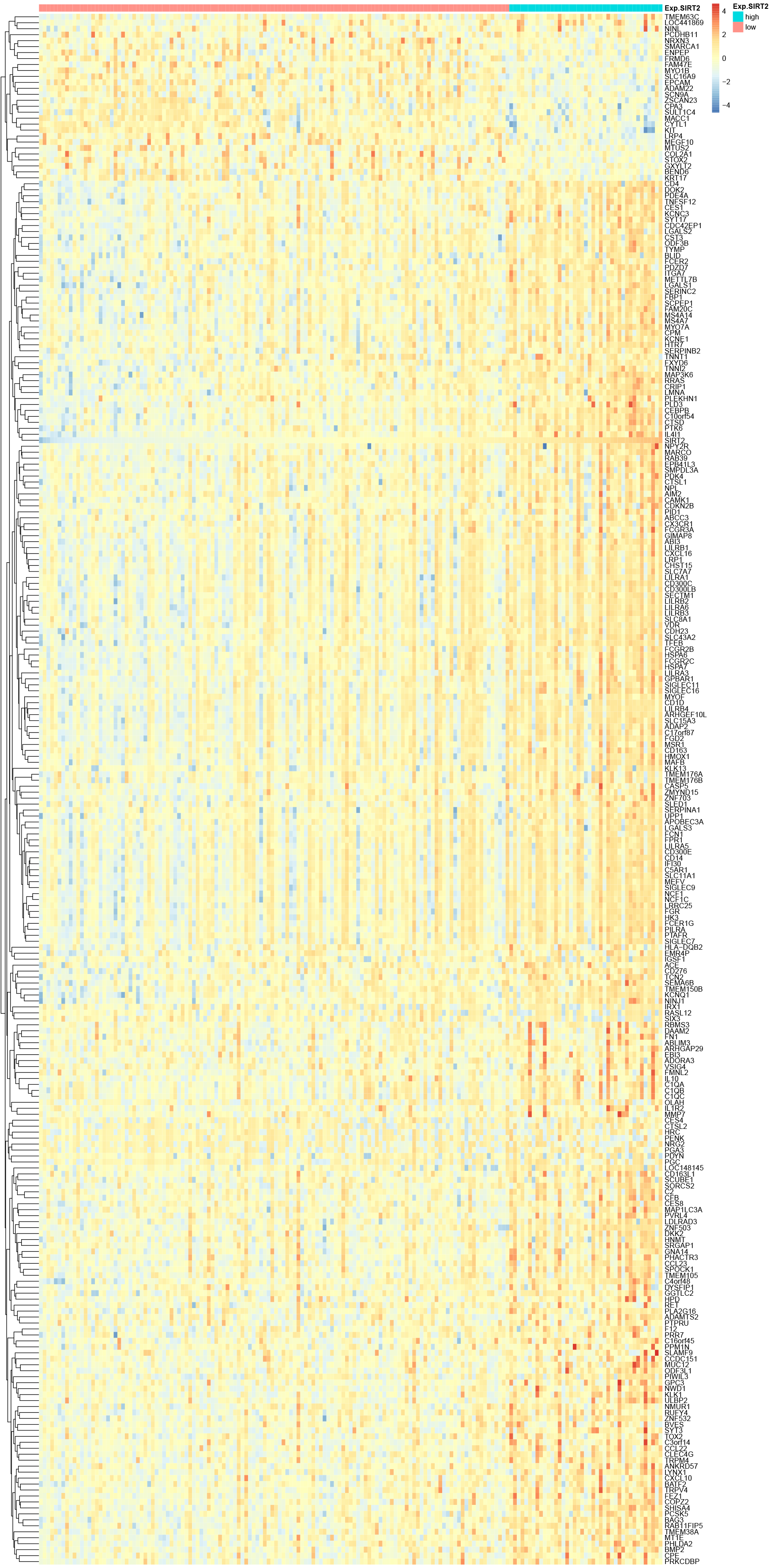
**

**Figure S1.** Heatmap of associated genes.

**
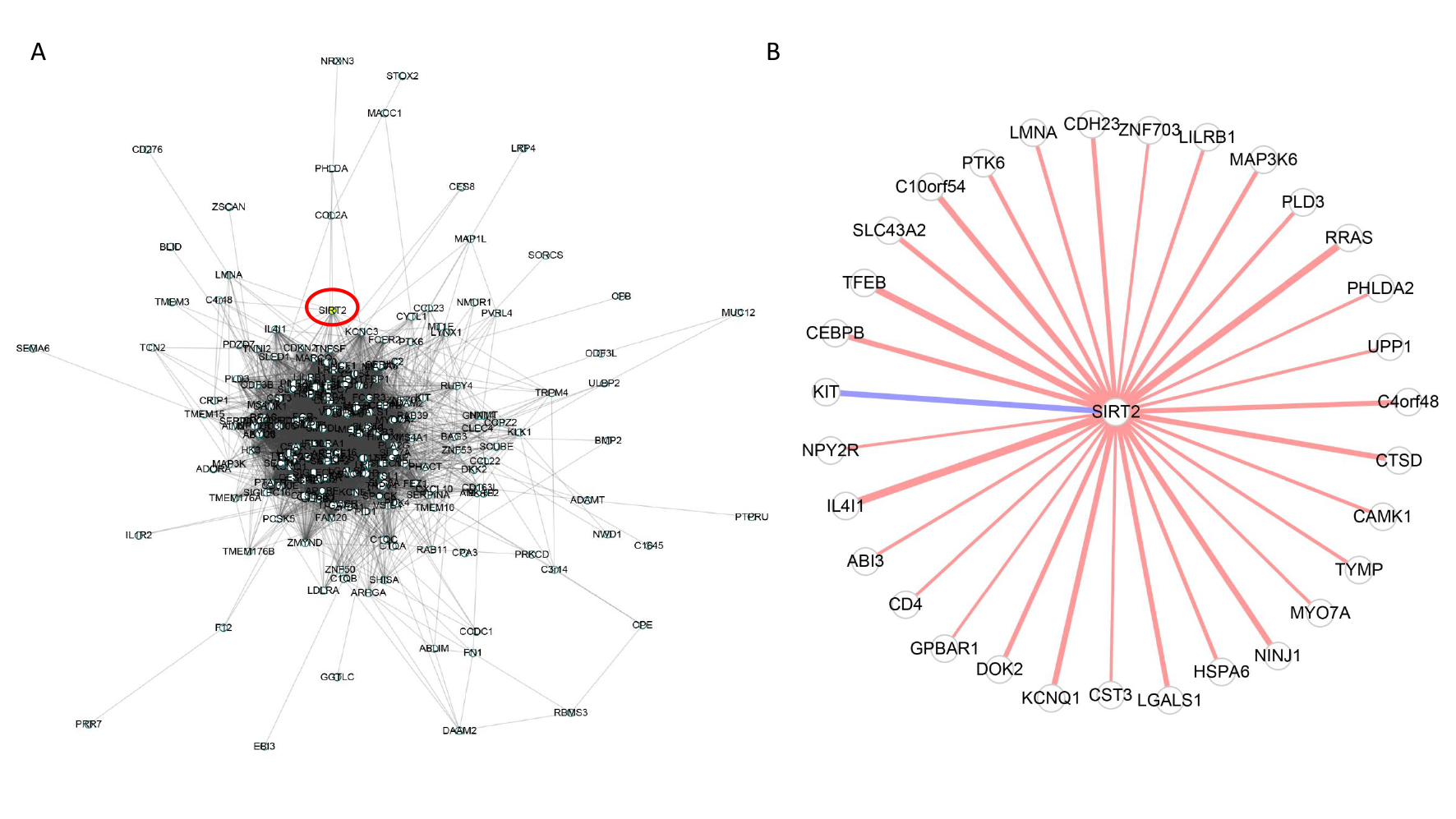
**

**Figure S2.** Molecular network of SIRT2. A. Molecular network of differential expressed genes and SIRT2. B. Correlated genes of SIRT2.

**
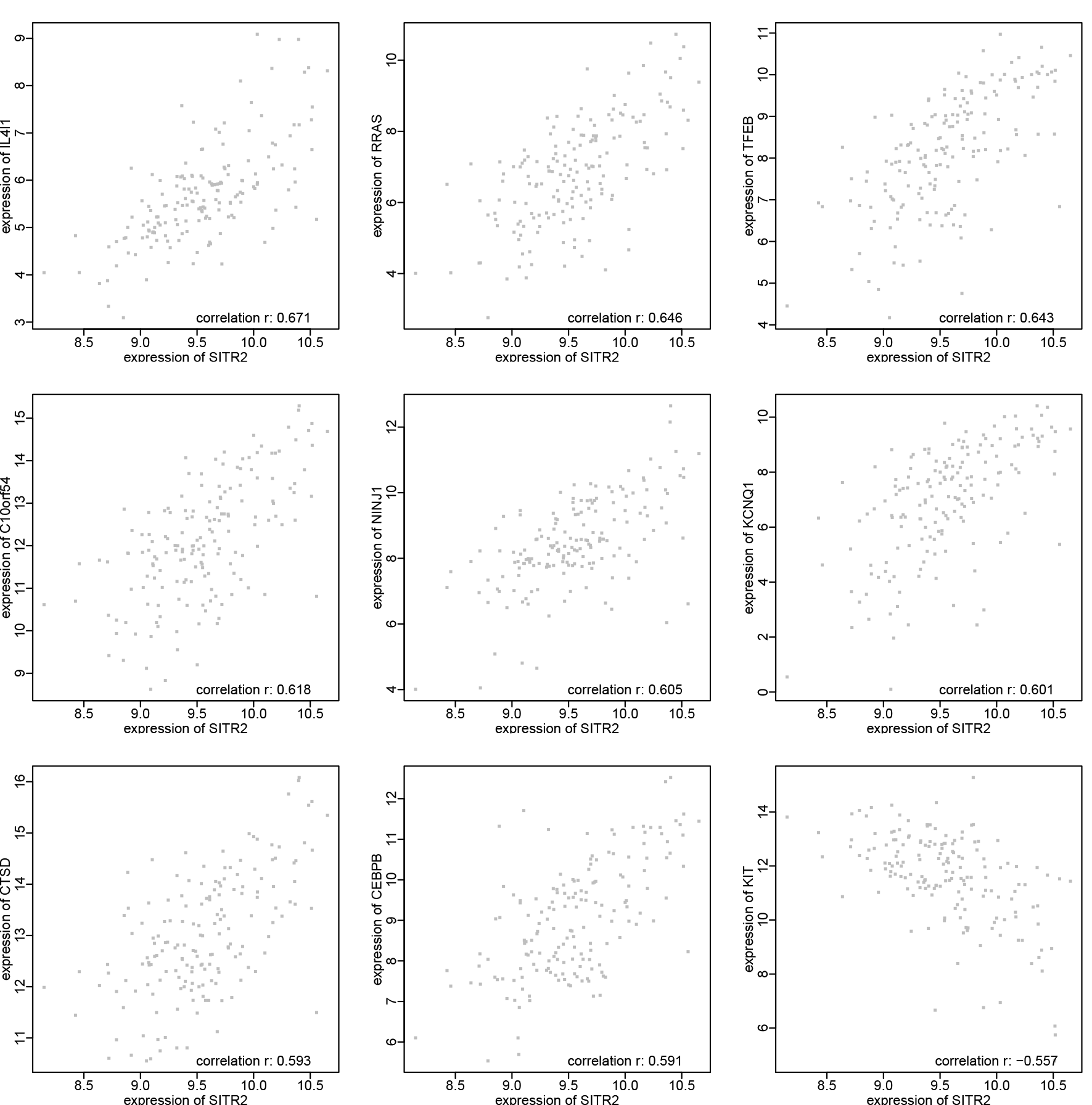
**

**Figure S3.** Scatter plot of the DEGs’ expression patterns with SIRT2.

**Table S1.** Upregulated differential genes associated with SIRT2 expression.

| geneNames | log2FC | p-Value |
| --- | --- | --- |
| NCF1C | 1.5050169 | 3.51E-05 |
| GIMAP8 | 1.508925605 | 1.52E-05 |
| PTAFR | 1.511777502 | 5.26E-08 |
| FCGR2B | 1.513038269 | 7.12E-05 |
| TMEM150B | 1.514084239 | 1.01E-05 |
| TFEB | 1.516553498 | 2.78E-15 |
| TNFSF12 | 1.516711917 | 4.82E-08 |
| PCSK5 | 1.517495155 | 0.005157673 |
| MS4A7 | 1.519112707 | 0.000149709 |
| LRP1 | 1.520307917 | 2.66E-05 |
| MT1E | 1.52111999 | 0.003371053 |
| SIGLEC9 | 1.523229475 | 6.60E-07 |
| CHST15 | 1.535249244 | 1.44E-05 |
| C16orf45 | 1.535650167 | 0.004309953 |
| LILRA1 | 1.539635025 | 1.52E-07 |
| CTSD | 1.542625415 | 1.25E-13 |
| ADORA3 | 1.543615194 | 0.000603188 |
| PDE4A | 1.544791445 | 4.30E-07 |
| FXYD6 | 1.549917894 | 0.000950028 |
| MEFV | 1.550070503 | 2.90E-06 |
| CDH23 | 1.550573463 | 1.85E-09 |
| SCPEP1 | 1.550837524 | 2.52E-09 |
| FMNL2 | 1.552405265 | 0.001384389 |
| GGTLC2 | 1.554458095 | 0.004269043 |
| VDR | 1.559978284 | 5.68E-11 |
| MAP3K6 | 1.562033927 | 2.52E-12 |
| CD1D | 1.565117466 | 2.81E-08 |
| NCF1 | 1.567683203 | 4.13E-06 |
| PILRA | 1.575813787 | 4.41E-09 |
| SLC43A2 | 1.576213644 | 4.11E-11 |
| ZNF532 | 1.578798901 | 2.38E-07 |
| PTK6 | 1.579219693 | 5.57E-05 |
| FCER1G | 1.581944679 | 2.90E-08 |
| SLED1 | 1.590120939 | 0.000216342 |
| CST3 | 1.59376597 | 5.20E-13 |
| BATF2 | 1.596740077 | 0.006036333 |
| FCN1 | 1.603613781 | 6.31E-05 |
| EMR4P | 1.604588845 | 0.003235248 |
| GNA14 | 1.605785213 | 0.005076267 |
| CDC42EP1 | 1.608080952 | 0.000385139 |
| PRR7 | 1.612760167 | 0.000437003 |
| PDZD7 | 1.619532012 | 0.002420313 |
| CD300LB | 1.622414561 | 4.08E-08 |
| FGR | 1.623853684 | 1.72E-10 |
| DKK2 | 1.63188756 | 0.001239558 |
| BAG3 | 1.634152506 | 2.98E-06 |
| NMUR1 | 1.634896137 | 0.005926164 |
| PTPRU | 1.636242292 | 0.001328305 |
| FCER2 | 1.638006401 | 9.68E-06 |
| RAB39 | 1.646669312 | 0.003379001 |
| MYOF | 1.649066711 | 5.36E-05 |
| LGALS2 | 1.651640233 | 0.000411615 |
| KCNQ1 | 1.656647928 | 3.46E-13 |
| SLC7A7 | 1.656704925 | 4.39E-05 |
| CD4 | 1.65741437 | 8.63E-12 |
| AIM2 | 1.657809231 | 0.002550186 |
| FPR1 | 1.659103021 | 7.14E-05 |
| MAP1LC3A | 1.665341991 | 0.000418491 |
| HNMT | 1.669759925 | 8.83E-07 |
| TMEM176A | 1.678113419 | 0.000617371 |
| NINJ1 | 1.680622222 | 5.26E-09 |
| ACE | 1.680855517 | 0.002222152 |
| LMNA | 1.684992419 | 3.15E-10 |
| CD276 | 1.691484474 | 1.45E-06 |
| UPP1 | 1.697436904 | 5.22E-09 |
| DOK2 | 1.698222343 | 1.93E-13 |
| F12 | 1.699116651 | 0.000564293 |
| IL4I1 | 1.705623296 | 1.87E-05 |
| LGALS3 | 1.706371534 | 5.97E-09 |
| LGALS1 | 1.706852646 | 1.42E-13 |
| ADAP2 | 1.709610497 | 1.84E-06 |
| CEBPB | 1.711641413 | 2.92E-14 |
| C4orf48 | 1.712698667 | 0.002016613 |
| FGD2 | 1.716856744 | 2.98E-10 |
| C10orf54 | 1.725240063 | 2.92E-16 |
| C17orf87 | 1.732581368 | 1.26E-06 |
| ABCC3 | 1.739445415 | 0.002299103 |
| TMEM38A | 1.742095951 | 0.00472725 |
| TNNI2 | 1.749013403 | 0.00103245 |
| SLC8A1 | 1.750538219 | 1.87E-06 |
| PLD3 | 1.753215095 | 5.19E-07 |
| HLA-DQB2 | 1.759420501 | 0.002321508 |
| NPL | 1.76076327 | 1.25E-05 |
| ZNF503 | 1.762460861 | 4.84E-11 |
| LOC441869 | 1.769711641 | 0.00629709 |
| CRIP1 | 1.774970815 | 2.09E-06 |
| SLC11A1 | 1.776628883 | 1.10E-07 |
| CD300C | 1.784214653 | 1.13E-12 |
| LILRB4 | 1.787647121 | 5.79E-08 |
| SERPINB2 | 1.794870054 | 0.00303067 |
| SERPINA1 | 1.795727292 | 8.60E-07 |
| ABI3 | 1.799197921 | 1.01E-07 |
| NINL | 1.81402381 | 0.004341372 |
| LRRC25 | 1.825203363 | 1.15E-09 |
| SLC15A3 | 1.841045681 | 1.79E-09 |
| APOBEC3A | 1.842545909 | 6.71E-06 |
| CD14 | 1.843018097 | 4.84E-06 |
| TMEM176B | 1.845022849 | 0.000392475 |
| PPM1N | 1.849123394 | 0.004699426 |
| MS4A14 | 1.849470853 | 3.62E-07 |
| CXCL10 | 1.850836223 | 0.002636078 |
| LILRA6 | 1.852187759 | 1.21E-06 |
| LILRA5 | 1.852719381 | 1.42E-05 |
| TCN2 | 1.859748183 | 6.84E-07 |
| ARHGEF10L | 1.861196278 | 1.99E-10 |
| FBP1 | 1.864180575 | 6.85E-07 |
| CX3CR1 | 1.875130113 | 7.06E-05 |
| PVRL4 | 1.882355777 | 0.003832395 |
| ODF3B | 1.89470479 | 2.50E-05 |
| ARHGAP29 | 1.900242816 | 0.002532494 |
| HTR7 | 1.900284307 | 8.89E-08 |
| IFI30 | 1.909408809 | 7.15E-09 |
| PLA2G16 | 1.927033758 | 0.000320112 |
| CES8 | 1.93744828 | 1.13E-05 |
| FAM20C | 1.943569029 | 9.19E-08 |
| LILRB3 | 1.946580132 | 4.26E-10 |
| CXCL16 | 1.956932144 | 0.000266546 |
| SIGLEC7 | 1.959167864 | 2.77E-10 |
| PHLDA2 | 1.959460036 | 0.0037652 |
| RRAS | 1.962853825 | 4.91E-12 |
| TOX2 | 1.974248513 | 0.003355393 |
| PLEKHN1 | 1.97700341 | 0.002629947 |
| CDKN2B | 1.981123875 | 2.35E-05 |
| RAB11FIP5 | 1.982607853 | 8.79E-08 |
| HSPA6 | 1.991460699 | 1.16E-06 |
| RET | 1.995168886 | 0.004963532 |
| BMP2 | 1.998156731 | 0.001899157 |
| LILRB1 | 1.998901219 | 3.21E-06 |
| RUFY4 | 2.003124831 | 0.000415126 |
| GPBAR1 | 2.004556448 | 1.28E-06 |
| COPZ2 | 2.008961241 | 0.00164773 |
| PRKCDBP | 2.022910415 | 0.001804726 |
| CPM | 2.034148044 | 1.96E-06 |
| CD300E | 2.034496179 | 6.05E-05 |
| CAMK1 | 2.039310992 | 6.62E-13 |
| C5AR1 | 2.039485152 | 6.60E-06 |
| HK3 | 2.045701561 | 2.21E-10 |
| CCL23 | 2.052825147 | 4.49E-06 |
| ODF3L1 | 2.055222439 | 0.005752548 |
| CCL22 | 2.065918431 | 0.003811257 |
| LILRB2 | 2.07093345 | 7.06E-09 |
| TMEM105 | 2.086655925 | 2.24E-06 |
| SRGAP1 | 2.09847369 | 1.25E-06 |
| DYSFIP1 | 2.100459069 | 0.000969497 |
| SIGLEC16 | 2.110322609 | 1.21E-05 |
| CTSL1 | 2.118042122 | 1.97E-06 |
| SEMA6B | 2.119911504 | 0.006194869 |
| KCNE1 | 2.146796247 | 5.30E-07 |
| METTL7B | 2.1482948 | 2.38E-05 |
| SCUBE1 | 2.152963884 | 3.38E-06 |
| TYMP | 2.153409212 | 2.37E-09 |
| ZMYND15 | 2.155601761 | 0.002980746 |
| HMOX1 | 2.176009269 | 3.55E-05 |
| ITGA7 | 2.177780619 | 5.74E-06 |
| FCGR2C | 2.180004177 | 0.000725234 |
| TNNT1 | 2.187065192 | 0.003898336 |
| SYT17 | 2.190495516 | 4.99E-05 |
| CES1 | 2.19360116 | 0.003729143 |
| CES4 | 2.196609035 | 0.00268719 |
| HPD | 2.221698036 | 0.002123691 |
| LDLRAD3 | 2.238005235 | 2.93E-09 |
| SYT3 | 2.240688745 | 0.001343408 |
| CD163L1 | 2.263103486 | 0.000125032 |
| ZNF703 | 2.268394546 | 2.39E-05 |
| SECTM1 | 2.285265269 | 3.74E-08 |
| SORCS2 | 2.288086249 | 1.82E-07 |
| CCDC151 | 2.29263092 | 0.004631623 |
| SHISA4 | 2.310705407 | 0.00024272 |
| SPOCK1 | 2.315755421 | 3.69E-07 |
| TMEM63C | 2.321977537 | 0.000246507 |
| BVES | 2.330126652 | 0.000466697 |
| PID1 | 2.34144862 | 0.000119957 |
| FN1 | 2.34453243 | 0.00066105 |
| SIGLEC11 | 2.35018861 | 4.96E-06 |
| CFB | 2.361291825 | 3.61E-06 |
| CASP5 | 2.36744468 | 0.006054465 |
| MUC12 | 2.371495066 | 0.003213579 |
| CPE | 2.378330721 | 0.001181007 |
| SLAMF9 | 2.395995639 | 0.006406881 |
| ULBP2 | 2.402076873 | 0.001433039 |
| EBI3 | 2.408246565 | 0.001585348 |
| NRG2 | 2.415845347 | 0.003411383 |
| SERINC2 | 2.421168829 | 3.10E-07 |
| ANKRD57 | 2.450989978 | 0.000170408 |
| IL10 | 2.453748748 | 0.001465523 |
| HSPA7 | 2.471255535 | 2.57E-05 |
| ADAMTS2 | 2.480538789 | 0.000270135 |
| RBMS3 | 2.484256627 | 0.000875522 |
| VSIG4 | 2.501608725 | 0.000199442 |
| LYNX1 | 2.506301696 | 3.15E-06 |
| SMPDL3A | 2.509580442 | 0.000107663 |
| MAFB | 2.561594379 | 2.87E-08 |
| NWD1 | 2.653129806 | 0.001738549 |
| CTSL2 | 2.683610904 | 0.000512248 |
| CLEC4G | 2.738873698 | 0.000190605 |
| MSR1 | 2.744578947 | 0.00304582 |
| MARCO | 2.755304764 | 0.005436742 |
| PHACTR3 | 2.802497583 | 4.57E-05 |
| PGC | 2.803406747 | 0.002195255 |
| FCGR3A | 2.804320072 | 0.0046497 |
| FEZ1 | 2.809808153 | 0.000107433 |
| MYO7A | 2.82509928 | 4.03E-12 |
| LILRA3 | 2.839494898 | 1.60E-05 |
| KCNC3 | 2.848588569 | 6.63E-05 |
| C2 | 2.881996896 | 1.30E-07 |
| KLK1 | 2.885627704 | 3.28E-05 |
| ABLIM3 | 2.936340007 | 0.000744737 |
| TRPM4 | 2.960859773 | 0.000425427 |
| C1QC | 3.011602823 | 0.004799785 |
| PGA3 | 3.167282663 | 0.002393045 |
| EPB41L3 | 3.214003878 | 8.66E-06 |
| TRPV4 | 3.250526211 | 0.002178202 |
| PDK4 | 3.309889355 | 0.000132369 |
| C1QB | 3.373081641 | 0.005005075 |
| GPC3 | 3.393076753 | 0.001080144 |
| IGSF1 | 3.445388107 | 0.001498602 |
| CD163 | 3.561488421 | 9.87E-06 |
| IL1R2 | 3.623683671 | 0.006203868 |
| C3orf14 | 3.719648017 | 0.000246642 |
| HRC | 3.806837093 | 0.005949695 |
| C1QA | 3.862825072 | 0.000317425 |
| PDYN | 3.869861196 | 0.005215906 |
| DAAM2 | 3.977343534 | 0.003409925 |
| PENK | 4.063924991 | 0.001298943 |
| KLK13 | 4.107743403 | 0.002531135 |
| OLAH | 4.114733289 | 0.001038967 |
| MMP7 | 4.229849603 | 0.00333165 |
| NPY2R | 12.08246933 | 0.003704686 |

**Table S2.** Downregulated differential genes associated with SIRT2 expression.

| Gene | log2FC | p-Value |
| --- | --- | --- |
| RASL12 | -5.405501796 | 0.001090764 |
| LOC148145 | -4.962723703 | 0.000832163 |
| SIX3 | -4.847227885 | 0.000203568 |
| COL2A1 | -4.655230055 | 0.005164795 |
| BLID | -3.534229182 | 0.005254552 |
| PIWIL3 | -3.477324925 | 0.004564633 |
| NRXN3 | -3.210516088 | 0.005535362 |
| GXYLT2 | -3.188924578 | 0.004195274 |
| IRX1 | -2.993105437 | 0.001797772 |
| STOX2 | -2.925621846 | 0.000168251 |
| PCDHB11 | -2.810565533 | 0.006592041 |
| LRP4 | -2.796610753 | 0.00110506 |
| ZSCAN23 | -2.68656398 | 1.80E-06 |
| MEGF10 | -2.651112388 | 0.00175943 |
| FAM47E | -2.648920247 | 0.000519298 |
| SCN9A | -2.334257646 | 0.005730674 |
| MYO1B | -2.247312672 | 2.10E-05 |
| KRT17 | -2.208918342 | 0.006707606 |
| SMARCA1 | -2.09413909 | 0.000921262 |
| EPCAM | -2.055786796 | 0.0007149 |
| CYTL1 | -1.945445389 | 0.000992195 |
| SLC16A9 | -1.791042919 | 0.000782697 |
| CPA3 | -1.790537688 | 0.000136523 |
| BEND6 | -1.758786543 | 0.000303981 |
| FRMD6 | -1.72355929 | 0.000314717 |
| MTUS2 | -1.692342759 | 0.005580151 |
| ENPEP | -1.690997039 | 0.004024543 |
| MACC1 | -1.652649823 | 9.77E-06 |
| KIT | -1.645269674 | 7.63E-10 |
| SULT1C4 | -1.585980282 | 5.94E-05 |
| ADAM22 | -1.543064486 | 0.004395892 |

Table S3. List of correlated genes of SIRT2.

|  | Correlation coefficient | P value |
| --- | --- | --- |
| SIRT2 | 1 | 0 |
| IL4I1 | 0.670846 | 0 |
| RRAS | 0.646084 | 0 |
| TFEB | 0.642943 | 0 |
| C10orf54 | 0.617701 | 0 |
| NINJ1 | 0.605239 | 0 |
| KCNQ1 | 0.600743 | 0 |
| CTSD | 0.593307 | 0 |
| CEBPB | 0.590725 | 0 |
| SLC43A2 | 0.578589 | 2.22E-16 |
| LGALS1 | 0.578361 | 6.56E-12 |
| C4orf48 | 0.575682 | 1.31E-11 |
| DOK2 | 0.567796 | 3.89E-11 |
| CDH23 | 0.560217 | 1.03E-10 |
| MAP3K6 | 0.557102 | 1.54E-10 |
| PTK6 | 0.554126 | 2.30E-10 |
| HSPA6 | 0.545019 | 7.53E-10 |
| PLD3 | 0.542434 | 1.05E-09 |
| LMNA | 0.537899 | 1.86E-09 |
| LILRB1 | 0.531197 | 4.27E-09 |
| CAMK1 | 0.526867 | 7.24E-09 |
| TYMP | 0.525648 | 3.01E-13 |
| MYO7A | 0.515476 | 2.80E-08 |
| ABI3 | 0.51505 | 2.94E-08 |
| UPP1 | 0.513535 | 1.28E-12 |
| CD4 | 0.512286 | 4.05E-08 |
| PHLDA2 | 0.512089 | 4.15E-08 |
| ZNF703 | 0.507815 | 2.48E-12 |
| GPBAR1 | 0.504603 | 9.72E-08 |
| NPY2R | 0.502249 | 1.26E-07 |
| CST3 | 0.501309 | 1.40E-07 |
| KIT | -0.55653 | 1.67E-10 |

Table S4. Gene sets upregulated in SIRT2^high^ group.

| NAME | NES | NOM p-value | FDR q-value |
| --- | --- | --- | --- |
| REGULATION OF ACTIN CYTOSKELETON | 1.816627 | 0 | 0.134686 |
| FRUCTOSE AND MANNOSE METABOLISM | 1.814478 | 0 | 0.109322 |
| FC EPSILON RI SIGNALING PATHWAY | 1.71143 | 0 | 0.093055 |
| MAPK SIGNALING PATHWAY | 1.639836 | 0 | 0.117102 |
| NEUROTROPHIN SIGNALING PATHWAY | 1.83169 | 0.002141 | 0.160225 |
| LYSOSOME | 1.945258 | 0.002155 | 0.129545 |
| CHEMOKINE SIGNALING PATHWAY | 1.7836 | 0.002193 | 0.093314 |
| B CELL RECEPTOR SIGNALING PATHWAY | 1.78248 | 0.003953 | 0.084942 |
| VEGF SIGNALING PATHWAY | 1.743939 | 0.004124 | 0.086556 |
| ENDOCYTOSIS | 1.794895 | 0.00432 | 0.095631 |
| GNRH SIGNALING PATHWAY | 1.622067 | 0.004608 | 0.108214 |
| ADIPOCYTOKINE SIGNALING PATHWAY | 1.688744 | 0.004728 | 0.106689 |
| FC GAMMA R MEDIATED PHAGOCYTOSIS | 1.846101 | 0.006369 | 0.207785 |
| PRION DISEASES | 1.762328 | 0.007317 | 0.094991 |
| ACUTE MYELOID LEUKEMIA | 1.737859 | 0.008333 | 0.084663 |
| LEUKOCYTE TRANSENDOTHELIAL  MIGRATION | 1.682687 | 0.008475 | 0.095242 |
| LEISHMANIA INFECTION | 1.796307 | 0.008621 | 0.10951 |
| VIBRIO CHOLERAE INFECTION | 1.759245 | 0.008696 | 0.089027 |
| SPHINGOLIPID METABOLISM | 1.638488 | 0.009091 | 0.108551 |
| CHRONIC MYELOID LEUKEMIA | 1.621289 | 0.010438 | 0.105095 |
| EPITHELIAL CELL SIGNALING IN  HELICOBACTER PYLORI INFECTION | 1.684775 | 0.011013 | 0.098367 |
| GLYCEROPHOSPHOLIPID METABOLISM | 1.608691 | 0.011521 | 0.107076 |
| VASOPRESSIN REGULATED  WATER REABSORPTION | 1.555126 | 0.013483 | 0.108139 |
| PATHOGENIC ESCHERICHIA  COLI INFECTION | 1.732752 | 0.014433 | 0.083892 |
| VIRAL MYOCARDITIS | 1.755638 | 0.014957 | 0.084358 |
| MELANOGENESIS | 1.635867 | 0.015945 | 0.106228 |
| INSULIN SIGNALING PATHWAY | 1.611631 | 0.016427 | 0.108626 |
| AMYOTROPHIC LATERAL SCLEROSIS ALS | 1.565794 | 0.016746 | 0.113693 |
| GLYCEROLIPID METABOLISM | 1.593242 | 0.018018 | 0.107885 |
| TOLL LIKE RECEPTOR  SIGNALING PATHWAY | 1.657013 | 0.021186 | 0.112802 |
| GLYCOSPHINGOLIPID BIOSYNTHESIS  GANGLIO SERIES | 1.635035 | 0.021834 | 0.102601 |
| AXON GUIDANCE | 1.44323 | 0.022059 | 0.157769 |
| APOPTOSIS | 1.565042 | 0.025 | 0.111216 |
| ALZHEIMERS DISEASE | 1.686374 | 0.025478 | 0.103154 |
| NOTCH SIGNALING PATHWAY | 1.547702 | 0.027311 | 0.108821 |
| NATURAL KILLER CELL MEDIATED  CYTOTOXICITY | 1.638893 | 0.02863 | 0.112842 |
| PROSTATE CANCER | 1.441613 | 0.034335 | 0.151323 |
| NOD LIKE RECEPTOR  SIGNALING PATHWAY | 1.560702 | 0.035865 | 0.111933 |
| GLYCOSAMINOGLYCAN BIOSYNTHESIS  CHONDROITIN SULFATE | 1.581696 | 0.037363 | 0.107309 |
| GALACTOSE METABOLISM | 1.64987 | 0.037736 | 0.112802 |
| ENDOMETRIAL CANCER | 1.492366 | 0.041068 | 0.130912 |
| RENAL CELL CARCINOMA | 1.470262 | 0.045267 | 0.143966 |
| MTOR SIGNALING PATHWAY | 1.425352 | 0.045952 | 0.157411 |
| GLYCOSAMINOGLYCAN DEGRADATION | 1.576236 | 0.047619 | 0.10813 |
| CARDIAC MUSCLE CONTRACTION | 1.600832 | 0.048055 | 0.109634 |
| GLUTATHIONE METABOLISM | 1.55185 | 0.049569 | 0.107955 |

Table S5. List of differentially methylated genes.

| Position | Gene | Βvalue(SIRT2^high^)  –Βvalue(SIRT2^low^) |
| --- | --- | --- |
| intronic | UBR4 | -0.22984 |
| intronic | TSNARE1 | 0.220157 |
| intronic | TNS3 | -0.22012 |
| intergenic | MYCN,FAM49A | -0.21554 |
| upstream | SDPR | 0.211219 |
| intergenic | CYSLTR2,FNDC3A | 0.208157 |
| intergenic | LOC101928767,LOC283585 | 0.207197 |
| intronic | BTBD9 | 0.207068 |
| intronic | MFSD2B | -0.20698 |
| UTR3 | ATF6 | 0.206969 |
| ncRNA_intronic | LOC101927066 | 0.201707 |
| intronic | HK2 | -0.2318 |
| UTR3 | EFCC1 | -0.2091 |
| ncRNA_intronic | MIR124-2HG | 0.202321 |
| intronic | EIF4H | 0.202228 |
| intronic | MAD1L1 | 0.201197 |
| intronic | PRDM16 | -0.22034 |
| intronic | LAMC3 | -0.21208 |
| ncRNA_intronic | LOC101927181 | 0.211971 |
| exonic | GNE | 0.202227 |
| upstream | C20orf141 | -0.20185 |
| intronic | ST6GAL1 | 0.201033 |
| intronic | MLKL | 0.222222 |
| ncRNA_intronic | LINC01483 | 0.218254 |
| intergenic | LINC00501,LINC00578 | 0.214068 |
| intronic | STAT1 | -0.20855 |
| intronic | BRAF | 0.205245 |
| intronic | METTL9 | 0.200308 |
| upstream;downstream | CEBPB-AS1;CEBPB | 0.22076 |
| intronic | COMMD3,COMMD3-BMI1 | 0.219823 |
| intronic | SNX29 | 0.219075 |
| UTR5 | IRF6 | 0.209195 |
| upstream;downstream | MARVELD2;RAD17 | 0.204217 |
| UTR3 | KBTBD11 | 0.20249 |
| intronic | GPRIN3 | 0.201124 |

**Table S6.** KEGG pathway analysis of differentially methylated genes.

| KEGG pathway | p-Value |
| --- | --- |
| Amino sugar and nucleotide sugar metabolism | 0.005247 |
| Butirosin and neomycin biosynthesis | 0.013426 |
| TNF signaling pathway | 0.026388 |
| Toxoplasmosis | 0.030892 |
